# Serum and plasma levels of Ba, but not those of soluble C5b-9, might be affected by renal function in chronic kidney disease patients

**DOI:** 10.1186/s12882-022-03022-z

**Published:** 2023-02-02

**Authors:** Ryoko Yamane, Yoshinari Yasuda, Aki Oshima, Yasuhiro Suzuki, Hiroshi Kojima, Hangsoo Kim, Sosuke Fukui, Shoichi Maruyama, Yasuhiko Ito, Masashi Mizuno

**Affiliations:** 1 Nephrology, Nagoya University Graduate School of Medicine, 65 Tsurumai-Cho, Showa-Ku, Nagoya, Japan; 2 Department of Renal Replacement Therapy, Nagoya University Graduate School of Medicine, 65 Tsurumai-Cho, Showa-Ku, Nagoya, Japan; 3 Department of Nephrology and Rheumatology, Aichi Medical University, Nagakute, Japan

**Keywords:** Chronic kidney disease, Complement, Ba, C5a, sC5b-9, Inulin clearance

## Abstract

**Background:**

During the last few decades, pathogenic mechanisms associated with uncontrolled activation of the complement (C) system and development of anti-C agents have been closely investigated in the field of nephrology. The usefulness of some C products such as C5a and sC5b-9 for diagnostic and prognostic purposes remains controversial. On the other hand, decreased renal function is being observed in many patients with or without nephritis as a background factor in progressively aging societies. We therefore investigated whether renal function influenced the evaluation of various complement components and activation products.

**Methods:**

To investigate the influence of renal function on evaluations of C3, C4, CH50, Ba, C5a and sC5b-9, 40 patients were retrospectively chosen from among 844 patients without active glomerulonephritis from 2009 to 2016. We measured plasma and serum levels of C3, C4, CH50, Ba, C5a and sC5b-9 using enzyme-linked immunosorbent assays and compared the findings with inulin clearance (Cin) as a marker of preserved renal function.

**Results:**

Both plasma and serum levels of Ba correlated significantly with Cin, but other values did not. Compared with patients with Cin ≥ 60 or ≥ 30 mL/min/1.73 m^2^, plasma and serum levels of Ba were increased in patients with Cin decreased to < 60 or < 30 mL/min/1.73 m^2^, but levels of C5a and sC5b-9 were not.

**Conclusion:**

The influence of renal function might need to be considered when evaluating Ba, but not C5a and sC5b-9, in plasma and serum samples from chronic kidney disease patients.

**Supplementary Information:**

The online version contains supplementary material available at 10.1186/s12882-022-03022-z.

## Background

The complement (C) system plays roles as an important innate immune system and represents a bridge to the acquired immune system in the host. The C system is generally maintained under as a balance between inhibition and activation in the host [1, 2]. However, the effects of the C system sometimes represent a double-edged sword, as impaired regulation and/or excessive activation of the C system can develop and/or enhanced various pathogenic conditions such as atypical hemolytic uremic syndrome (aHUS) and C3 glomerulopathy (C3G) in the fields of nephrology and Guillain–Barre syndrome, myasthenia gravis and neuromyelitis optica [3, 4].

In recent decades, as C-dependent mechanisms have been clarified, the clinical efficacies of anti-C therapies have been receiving increasing attention. As a result, anti-C agents have either become clinically available (such as C1-inhibitor and anti-C5 antibody) or are under development in phase 2, 3 and preclinical trials (such as C5a receptor blocker, factor B inhibitor and other agents) [2, 5]. As the development of anti-C agents progresses, searches for biomarkers associated with the C system could also become important. As part of such investigations, examination of serum/plasma Ba, C5a and sC5b-9 levels may be useful to consider potential diagnoses and/or disease activities for some pathogenic conditions such as aHUS and C3G in the field of nephrology [6]. Although aHUS and C3G are mostly encountered in younger generations [7–10], some patients are categorized as having chronic kidney disease (CKD), such as adults with heminephrectomy or elderly patients with nephrosclerosis. When evaluating biomarkers, it remains unclear how results are influenced under conditions of renal impairment in adults with CKD, particularly older patients. When evaluating serum/plasma levels of C components and products in adults with CKD including aged patients, background renal function may need to be considered. The present study therefore investigated serum and plasma levels of C activation products and the relationship between these and renal function as evaluated by inulin clearance in CKD patients without active glomerulonephritis.

## Materials and methods

### Patients

The present study was performed with the approval of the Ethics Committee for Human Research of the Faculty of Medicine at Nagoya University (approval no. #2012–0232-3308) and all participants agreed to join the study. We selected 40 of 844 patients registered from May 2009 to April 2016 according to the following criteria. All selected patients were categorized as not showing active glomerulonephritis. Detailed inclusion criteria were: 1) nephrosclerosis caused by atherosclerosis; 2) heminephrectomy due to resection for renal cell cancer or due to donor status for renal transplantation; and 3) glomerulonephritis less than 1.0 urinary protein amount/urinary creatinine level (g-Cre) without cellular casts or dysmorphic red blood cells under urinary examination. Exclusion criteria were: 1) absence of laboratory data including urinary analysis, inulin clearance (Cin) and estimated glomerular filtration rate (eGFR) to evaluate renal function [11]; or 2) a lack of serum and plasma samples stored at -80 °C; or 3) glomerulonephritis, defined as urinary protein > 1.0 g-Cre, with either cellular casts or dysmorphic red blood cells apparent in urinary examination. In terms of sample collection, blood was harvested in a tube with a separation agent for serum or in a tube containing EDTA-2Na^+^ for plasma, immediately centrifuged, and then at -80℃ until usage. We also collected laboratory data from the medical records of each patient including Japanese adjusted eGFR calculated according to a previous report [12]. Background characteristics of participants in the present study are shown in Table 1.

**Table 1:** Basic characteristics of patients

Total number (n)	40
Age (years) (mean ± SD)	56.1 ± 11.3
Sex (male/female), n	24/16
Causes of CKD^a^%, n (%)	
Candidate donor for living renal transplant	10 (25.0%)
Heminephrectomy	10^b^ (25.0%)
Nephrosclerosis	8 (20.0%)
IgA nephropathy without active urine sedimentation	8 (20.0%)
Post-renal transplantation (recipient)	2 (5.0%)
Other	2^c^ (5.0%)
Grade (G) of CKD (n (%))	
G1	4 (10.0)
G2	11 (27.5)
G3	18 (45.0)
G4	6 (15.0)
G5	1 (2.5)

^a^Chronic kidney disease; ^b^3 donors for renal transplantation and 7 renal cell carcinomas and no recurrences after > 5 years; ^c^1 with polycystic kidney, 1 with post-renal renal failure

### Measurement of Cin

The measured GFR was determined by Cin calculated from serum (the average of two points), urine concentrations and urine flow rate, and expressed as mL/min/1.73 m^2^ of body surface area. Cin was measured by simple method as described below and we have previously reported that a single urine collection after 60 min offered sufficient accuracy [11]. Inulin (1%) was administered by continuous intravenous infusion for 105 min under conditions of hydrated overnight fasting. During inulin infusion, serum samples were collected twice, at 45 and 105 min after starting infusion, for creatinine and inulin. The urine sample for inulin and creatinine was collected at 105 min after starting infusion after completely emptying the bladder at 45 min after starting inulin infusion.

### Measurement of total complement hemolytic assay (CH50)

We measured CH50 in serum and plasma using a CH50 kit in accordance with the instructions from the manufacturer (Denka Company, Tokyo, Japan).

### Measurements of C components and C activation products (C3, C4, Ba, C5a and soluble C5b-9 (sC5b-9))

Enzyme-linked immunosorbent assays were used to investigate levels of C components and C activation products in serum and plasma samples. C3, C4, Ba, C5a and sC5b-9 were measured using N-assay TIA C3-SH Nittobo, N-assay TIA C4-SH Nittobo (Nittobo Medical Co., Koriyama, Japan), MicroVue Ba, C5a and sC5b-9 enzyme immunoassay kits (Quidel, San Diego, CA, USA), respectively. In each kit, the range of sample dilutions used is shown in Supplementary Table 1.

### Correlations between levels of C components or C activation products and renal function

We divided the 40 patients into groups using two different thresholds: Cin < 60 mL/min/1.73 m^2^ (Cin < 60 group) vs. Cin ≥ 60 mL/min/1.73 m^2^ (Cin ≥ 60 group); and Cin < 30 mL/min/1.73 m^2^ (Cin < 30 group) vs. Cin ≥ 30 mL/min/1.73 m^2^ (Cin ≥ 30 group). These groups were used to investigate correlations between levels of C3, C4, CH50, Ba, C5a and sC5b-9 and renal function. In the first set, Cin/eGFR < 30 mL/min/1.73 m^2^ was defined as CKD grade 4 and 5 (Cin < 30 group) and Cin/eGFR ≥ 30 mL/min/1.73 m^2^ as CKD grade 1–3 (Cin ≥ 30 group). In the second set, Cin/eGFR < 60 mL/min/1.73 m^2^ was defined as CKD grade 3–5 (Cin < 60 group) and Cin/eGFR ≥ 60 mL/min/1.73 m^2^ as CKD grade 1 and 2 (Cin ≥ 60 group).

### Statistical analysis

Correlations were evaluated using Spearman’s rank correlation coefficients. All values were compared between two groups using the Mann–Whitney U test. Data are expressed as mean ± standard deviation or median and interquartile range. Values of *p* < 0.05 were considered significant. Analyses were performed using IBM SPSS Statistics version 27 (IBM, Armonk, NY).

## Results

### Background characteristics and renal function of patients

Background characteristics of all patients are shown in Table 1. As the present study focused on CKD patients without active glomerulonephritis, decreased renal function in most cases was identified incidentally in candidate donors for living renal transplant, heminephrectomy for renal cell carcinoma or renal transplant donation, or nephrosclerosis. When we first evaluated correlations between Cin and eGFR in the present study, a significant correlation was observed between Cin and eGFR, as reported previously [12] (Supplementary Fig. 1).

### Comparisons of C3, C4, CH50, Ba, C5a, and sC5b-9 levels between serum and plasma

**Fig. 1:**
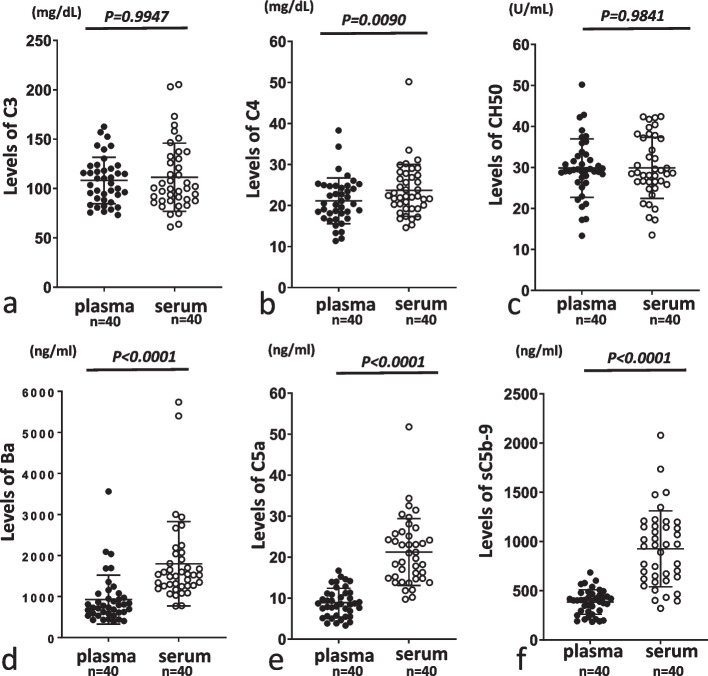
Comparison between plasma and serum levels of C3, C4, CH50, Ba, C5a and sC5b-9 and inulin clearance (Cin) as a marker of renal function. When we compared levels of C3 (**a**), C4 (**b**), CH50 (**c**), Ba as a fragment of factor B (**d**), C5a (**e**) and sC5b-9 (**f**) between plasma and serum samples, serum levels of C5a, Ba and sC5b-9 were significantly higher than plasma levels, although no differences were evident between plasma and serum levels of C3, C4 or CH50. Closed and open circle represent plasma and serum samples, respectively

No significant differences in levels of C3 or CH50 were observed between serum and plasma (Fig. 1a-c). Levels of Ba, C5a and sC5b-9 were clearly higher in serum than in plasma (Fig. 1d-f), as previously reported in a study of healthy Japanese volunteers [13]. Although serum C4 levels were also slightly but significantly higher than plasma levels, both levels were almost within standard ranges (Fig. 1b).

### Correlations between C3, C4, and CH50 in serum/plasma and renal function

**Fig. 2:**
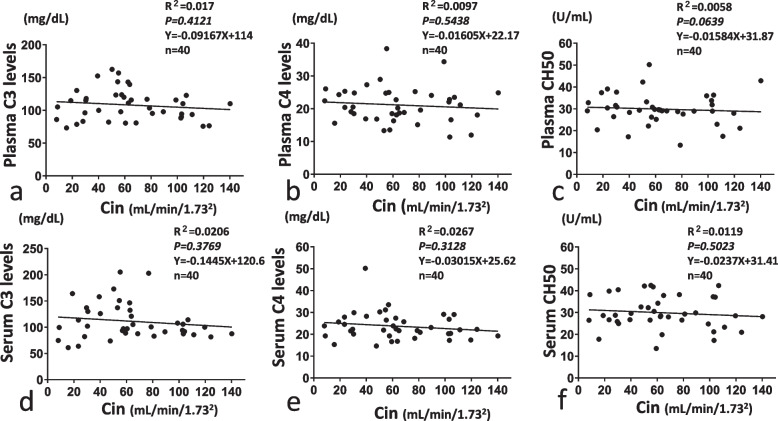
Correlation between plasma/serum levels of C3, C4, CH50 and inulin clearance (Cin). Correlations of levels of C3, C4 and CH50 with Cin are shown in **a** and **d**, **b** and **e**, and **c** and **f**, respectively. Correlations between plasma levels and Cin are shown in **a**, **b** and **c**, and correlations between serum levels and Cin are shown in **d**, **e** and **f**

First, we evaluated correlations between C3, C4 and CH50 as usual clinical findings and renal function as indicated by Cin or eGFR. No correlation between Cin and each serum or plasma level of C3 or C4 (as complement components) or CH50 (reflecting hemolytic activity) was observed (Fig. 2). Using eGFR as a reference instead of Cin, no correlations with either serum or plasma levels of C3, C4 or CH50 were seen (Supplementary Fig. 2).

### Correlation between C activation products Ba, C5a or sC5b-9 and renal function

**Fig. 3:**
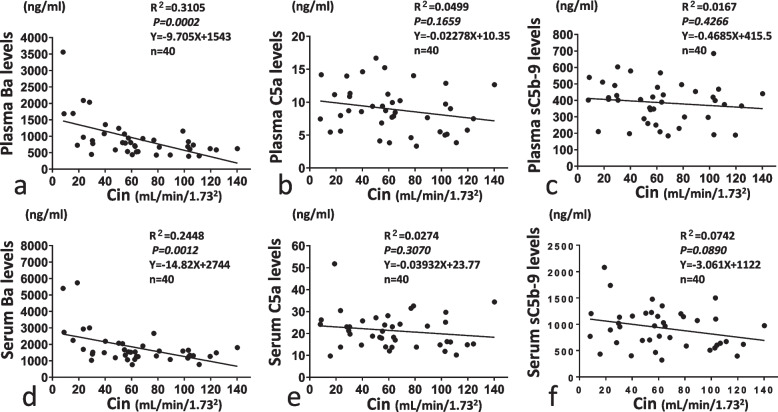
Correlation between plasma/serum levels of Ba, C5a, sC5b-9 and inulin clearance (Cin). Correlations of Ba, C5a and sC5b-9 levels with Cin are shown in **a** and **d**, **b** and **e**, and **c** and **f**, respectively. Correlations between plasma levels and Cin are shown in **a**, **b** and **c**, and correlations with serum levels and Cin are shown in **d**, **e** and **f**

When we investigated correlations between C activation products and Cin as a marker of renal function, a significant correlation between Cin and each of the serum and plasma levels of Ba was observed (Fig. 3a, d). In contrast, other C activation products such as serum/plasma levels of C5a and sC5b-9 showed no significant correlations with Cin (Fig. 3b, c, e, f). As the reference, eGFR also correlated with serum and plasma Ba levels, but not with serum or plasma levels of C5a or CH50 (Supplementary Fig. 3). We used Cin but not eGFR as the marker of renal function for the following analysis.

### Comparison of serum and plasma levels of C3, C4, CH50, Ba, C3a, C5a and sC5b-9 between the Cin < 60 and Cin ≥ 60 groups and between the Cin < 30 and Cin ≥ 30 groups

**Fig. 4:**
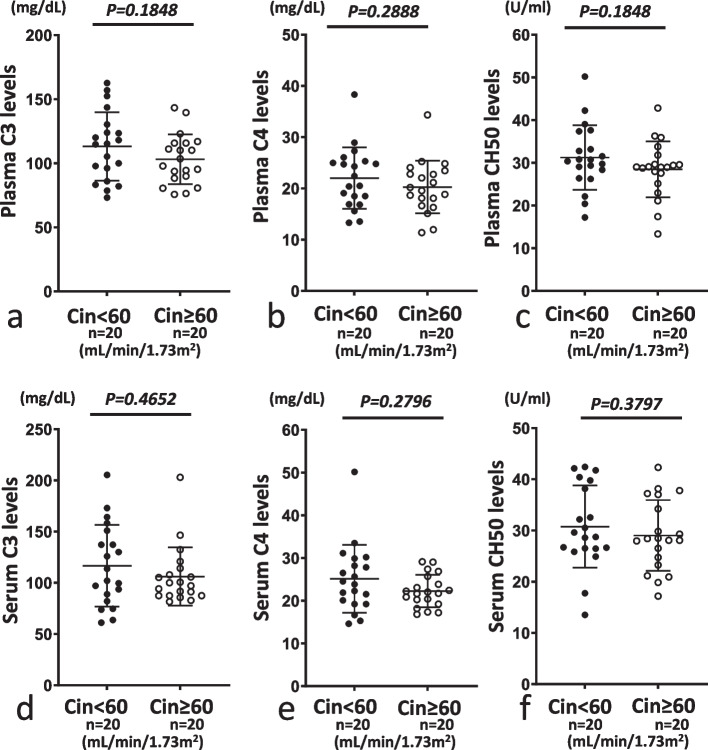
Comparison of plasma or serum levels of C3, C4 and CH50 between inulin clearance (Cin) < 60 mL/min/1.73 m^2^ (Cin < 60) and Cin ≥ 60 mL/min/1.73 m^2^ (Cin ≥ 60). In plasma and serum samples, levels of C3, C4 and CH50 did not differ significantly between Cin < 60 and Cin ≥ 60. **a** and **d**, **b** and **e**, and **c** and **f** show levels of C3, C4 and CH50, respectively. **a**–**c** and **d**–**f** show plasma and serum samples, respectively. Closed and open circles represent Cin < 60 and Cin ≥ 60, respectively

**Fig. 5:**
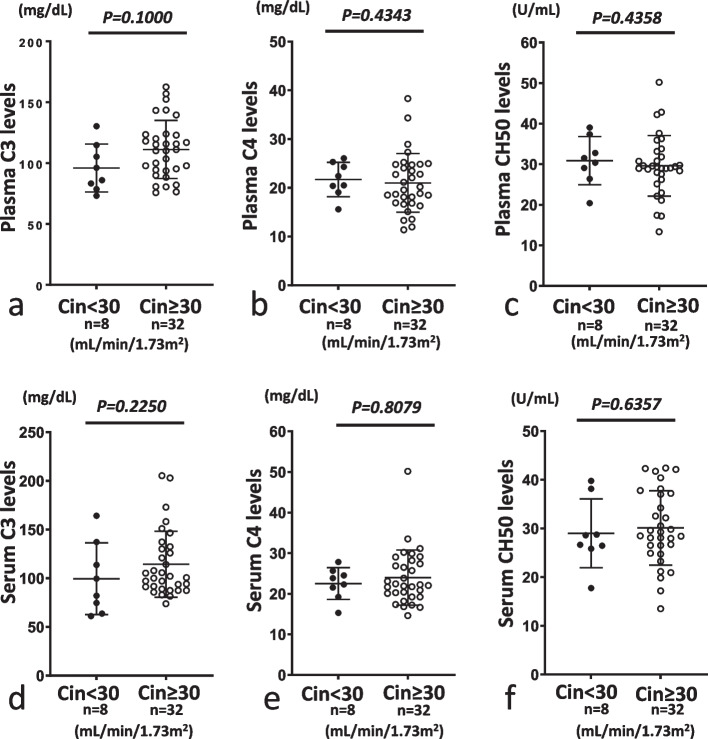
Comparison of serum or plasma levels of C3, C4 and CH50 between Cin < 30 mL/min/1.73 m^2^ (Cin < 30) and Cin ≥ 30 mL/min/1.73 m^2^ (Cin ≥ 30). In serum and plasma samples, levels of C3, C4 and CH50 did not differ significantly between Cin < 30 and Cin ≥ 30. **a** and **d**, **b** and **e**, and **c** and **f** show levels of C3, C4 and CH50, respectively. **a**–**c** and **d**–**f** show plasma and serum samples, respectively. Closed and open circles represent Cin < 30 and Cin ≥ 30, respectively

We then compared the two sets of groups (Cin < 60 vs. Cin ≥ 60 and Cin < 30 vs. Cin ≥ 30) and investigated the influence of decreased renal function. When we compared levels of C3, C4 and CH50 in individual serum and plasma samples, no significant differences were observed between either set of groups (Figs. 4, 5).

**Fig. 6:**
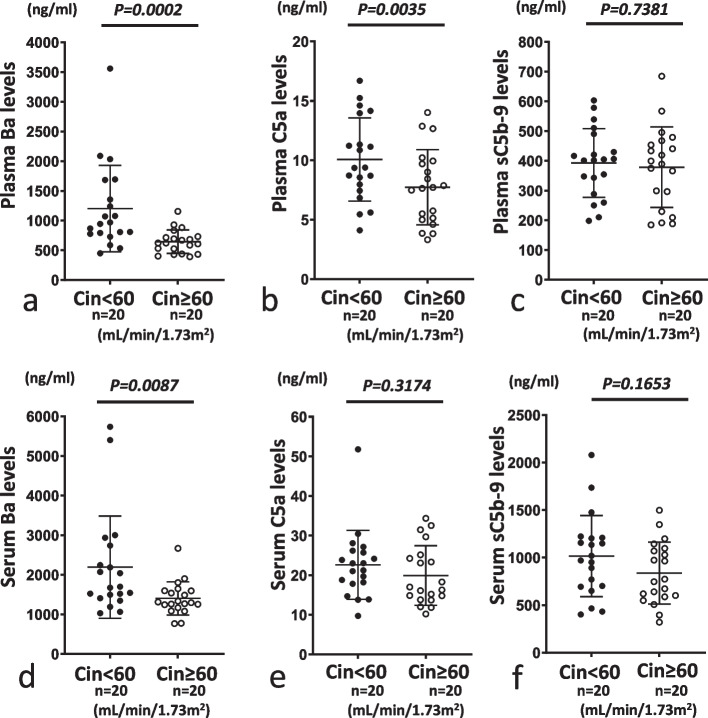
Comparison of plasma or serum levels of Ba, C5a and sC5b-9 between Cin < 60 mL/min/1.73 m^2^ (Cin < 60) and Cin ≥ 60 mL/min/1.73 m^2^ (Cin ≥ 60). For both plasma and serum samples, levels of Ba in Cin < 60 were significantly higher than those in Cin ≥ 60 (**a** and **d**). For plasma samples, levels of C5a were slightly but significantly higher in Cin < 60 than in Cin ≥ 60 (**b**). On the other hand, serum C5a levels and plasma and serum sC5b-9 levels did not differ significantly between Cin < 60 and Cin ≥ 60 (**c**, **e** and **f**). **a** and **d**, **b** and **e**, and **c** and **f** show levels of Ba, C5a and sC5b-9, respectively. **a**–**c** and **d**–**f** show plasma and serum samples, respectively. Closed and open circles represent Cin < 60 and Cin ≥ 60, respectively

**Fig. 7:**
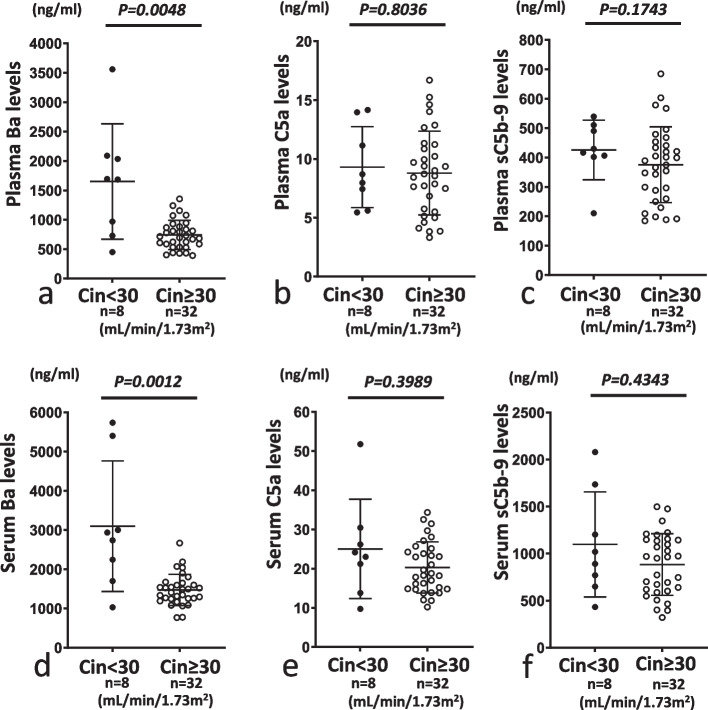
Comparison of serum or plasma levels of Ba, C5a and sC5b-9 between Cin < 30 mL/min/1.73 m^2^ (Cin < 30) and Cin ≥ 30 mL/min/1.73 m^2^ (Cin ≥ 30). For both plasma and serum samples, levels of Ba were significantly higher in Cin < 30 than in Cin ≥ 30 (**a** and **d**). On the other hand, plasma and serum levels of C5a and sC5b-9 did not differ significantly between Cin <30 and Cin ≥ 30 (**b**, **c**, **e** and **f**). **a** and **d**, **b** and **e**, and **c** and **f** show levels of Ba, C5a and sC5b-9, respectively. **a**–**c** and **d**–**f** show plasma and serum samples, respectively. Closed and open circles represent Cin < 30 and Cin ≥ 30, respectively

When we compared levels of Ba, C5a and sC5b-9 between sets of groups (Cin < 60 vs. Cin ≥ 60 and Cin < 30 vs. Cin ≥ 30) to evaluated C activation products, we observed significantly increased plasma and serum Ba levels and plasma C5a levels in Cin < 60 compared to Cin ≥ 60 and also significantly increased plasma and serum Ba levels in Cin < 30 compared to Cin ≥ 30 (Figs. 6a, b, d and 7a, d). However, no significant differences in serum and plasma levels of C5a or sC5b-9 were seen between Cin < 60 and Cin ≥ 60 or between Cin < 30 and Cin ≥ 30 (Figs. 6c, e, f and 7b, c, e, f).

## Discussion

Measuring systemic levels of C components and C activation products, in addition to studying C3, C4 and CH50 [14], may be useful to predict diagnosis, disease activity and/or prognosis for C-associated diseases, including some kidney diseases [3, 6]. However, as a background characteristic, renal function may be impaired in patients with CKD, particularly in elderly patients, and the effects on evaluations of C components and C activation products have remained unclear. We therefore investigated whether residual renal function may affect measurements of serum or plasma levels of C components and C activation products in CKD patients without active glomerulonephritis, excluding patients with end-stage renal disease (ESRD) on dialysis.

In the present study, both serum and plasma Ba levels correlated inversely with Cin and eGFR as markers of renal function, but neither serum nor plasma levels of C5a or sC5b-9 correlated with Cin or eGFR. In contrast, levels of C3, C4 and CH50 in both serum and plasma samples were within standard ranges and did not correlate with renal function. Concerning differences between plasma and serum levels, serum levels of Ba, C5a and sC5b-9 were significantly higher than plasma levels as in a previous report, although that study included only healthy subjects [13].

From our results associated with renal function according to CKD characterizations, we observed significant increases in serum and plasma levels of Ba in patients with Cin < 60 or Cin < 30 compared with Cin ≥ 60 or Cin ≥ 30, respectively. Although plasma C5a levels in Cin < 60 patients were also slightly but significantly higher than those in Cin ≥ 60 patients, the range of C5a levels was almost within the standard levels reported from a study of healthy Japanese individuals [13]. No other significant differences were identified between other pairs of groups. When measuring plasma and serum levels of Ba, C5a, sC5b-9, C3, C4 and CH50 under various pathological conditions, Ba as a fragment of factor B, a C activation product of the alternative pathway in complement activation system might be affected by background renal function such as elevation of factor D levels, which accelerated the alternative pathway [15–18]. However, other levels appear unaffected. Although C5a is a small molecule, C5a production might be suppressed because of several complement regulators at the level of C3 convertase formation [3].

The C system is a key system to maintain host heath and provide a strong defense against foreign materials [1, 3]. In contrast, uncontrolled activation of the C system can develop and enhance various pathological conditions [19, 20]. Maintaining a balance between activation and inhibition of the C system is considered important. In the field of nephrology, the C system is presently known to be associated with the pathogenic mechanisms underlying conditions such as aHUS and C3G and progression of renal injuries to both glomeruli and the interstitium in kidney diseases [3, 19, 21, 22]. In addition, development of anti-C agents has recently been proceeding. Some agents such as C1-inhibitors and anti-C5 antibodies have become clinically available and others with exact targets in the C system are in clinical or preclinical trials [2, 23, 24]. At the current stage of development of anti-C agents, development of biomarkers associated with the C system might also be much more important than a few decades ago. Moreover, C activation products were recently reported to assist in diagnosing and/or determining the severity of disease activity. In the field of nephrology, the usefulness of C5a and sC5b-9 is under discussion [24] and we have also tried to identify biomarkers in peritoneal dialysate to predict the prognosis of peritoneal dialysis-related peritonitis [25, 26], but these issues remain unclear.

Around the world, including in Japan, numbers of elderly patients and patients with CKD are increasing [27]. Renal function is reported to gradually decreased and fall to CKD in aged populations [28]. ESRD due to nephrosclerosis has therefore also increased in Japan in recent decades [29]. However, in most published reports investigating levels of C products, assessment of or adjustment for background renal function was not considered when evaluating the results, even in studies with aged patients. The present study evaluated GFR using Cin as well as eGFR, because Cin offers a more accurate method compared with creatinine clearance, and has usually been positioned as a gold standard for evaluating renal function [11]. Our results suggest that evaluation of Ba levels and plasma C5a levels should be considered with baseline renal function, whereas measurement of serum levels of sC5b-9 and serum C5a might not be clinically relevant in CKD in the absence of active glomerulonephritis. Why only serum and plasma levels of Ba showed correlations with renal function remains unclear. Hypothetically, because the molecular weights of Ba (~ 33 kDa) [30] are much smaller than those of C3, C4 and sC5b-9 (~ 190 kDa, ~ 187 kDa, and > 1000 kDa) [25, 31, 32], Ba might have more influence on decreased GFR than the others. Although C5a is also a small molecule (~ 11 kDa), C5a production might be suppressed because of several complement regulators at the level of C3 convertase formation [3]. As another hypothesis, an early stage of an alternative pathway in the C activation system may be constantly under ready-to-fire condition in a “tick-over” mechanism, resulting in Ba release [3] and it might be enhanced in patients with advanced CKD because of decreased blood pH and/or increased uremic toxins [33, 34]. However, later points of C regulation might be expected to effectively work to stop the terminal pathway of the C system. As a limitation to this study, the sample number was small and patients who had developed ESRD requiring dialysis therapies were not included. Whether the baseline levels of Ba in serum/plasma of CKD patients were adequate in this study remains unclear. In future, a larger-scale study is warranted to clarify this issue.

## Conclusion

In the present study, we observed that both serum and plasma levels of Ba increased depending on renal function without active glomerulonephritis, particularly in CKD G4 and G5 patients, but sC5b-9 was not affected by renal function in those patients. Among patients with advanced CKD, we suggest that Ba needs to be interpreted in the context of GFR when measuring and analyzing levels of C products in blood samples.

## Supplementary Information

**Additional file 1: Supplementary Fig. 1.** Correlation between eGFR and inulin clearance (Cin). A significant correlation was observed between eGFR and Cin in the present study. **Supplementary Fig. 2.** Correlation between plasma/serum levels of C3, C4, CH50 and estimated glomerular filtration rate (eGFR). This graph shows the correlation between plasma/serum levels and eGFR instead of inulin clearance (Cin). No significant correlation was observed between plasma/serum levels and eGFR, similar to Cin. **Supplementary Fig. 3.** Correlation between plasma/serum levels of Ba, C5a, sC5b-9 and eGFR. This graph shows the correlation between plasma/serum levels and eGFR instead of inulin clearance (Cin). No significant correlation was observed between plasma/serum levels and eGFR, similar to Cin.

**Additional file 2: Supplementary Table 1.** Sample dilutions for ELISA assays in this study.

## Data Availability

The datasets used and/or analyzed during the current study are available from the corresponding author on reasonable request.

## Abbreviations

C: Complement
aHUS: Atypical hemolytic uremic syndrome
C3G: C3 glomerulopathy
CKD: Chronic kidney disease
g-Cre: Urinary protein amount/urinary creatinine level
sC5b-9: Soluble C5b-9
Cin: Inulin clearance
eGFR: Estimated glomerular filtration rate
ESRD: End-stage renal disease
