# Supplementary material for: Serum and plasma levels of Ba, but not those of soluble C5b-9, might be affected by renal function in chronic kidney disease patients
Source: BMC Nephrol. 2023 Feb 2;24:26. doi: 10.1186/s12882-022-03022-z (PMC9893599; doi:10.1186/s12882-022-03022-z)
Supplement: Supplementary file 1 — Additional file 1: Supplementary Fig. 1. Correlation between eGFR and inulin clearance (Cin). A significant correlation was observed between eGFR and Cin in the present study. Supplementary Fig. 2. Correlation between plasma/serum levels of C3, C4, CH50 and estimated glomerular filtration rate (eGFR). This graph shows the correlation between plasma/serum levels and eGFR instead of inulin clearance (Cin). No significant correlation was observed between plasma/serum levels and eGFR, similar to Cin. Supplementary Fig. 3. Correlation between plasma/serum levels of Ba, C5a, sC5b-9 and eGFR. This graph shows the correlation between plasma/serum levels and eGFR instead of inulin clearance (Cin). No significant correlation was observed between plasma/serum levels and eGFR, similar to Cin. [file 12882_2022_3022_MOESM1_ESM.pptx]

## Slide 1
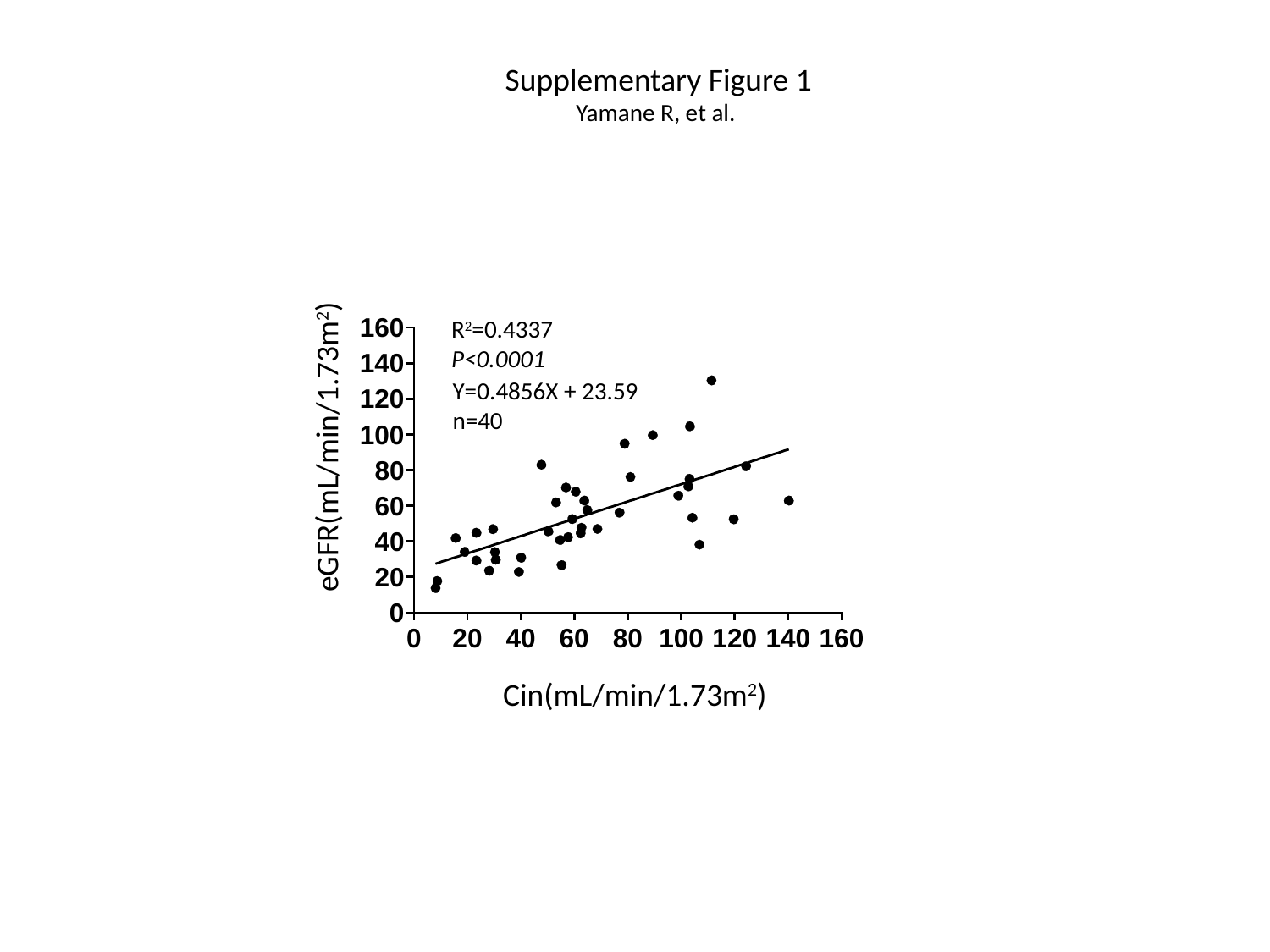

Supplementary Figure 1
Yamane R, et al.
R2=0.4337
P<0.0001
Y=0.4856X + 23.59
n=40
eGFR(mL/min/1.73m2)
Cin(mL/min/1.73m2)

## Slide 2
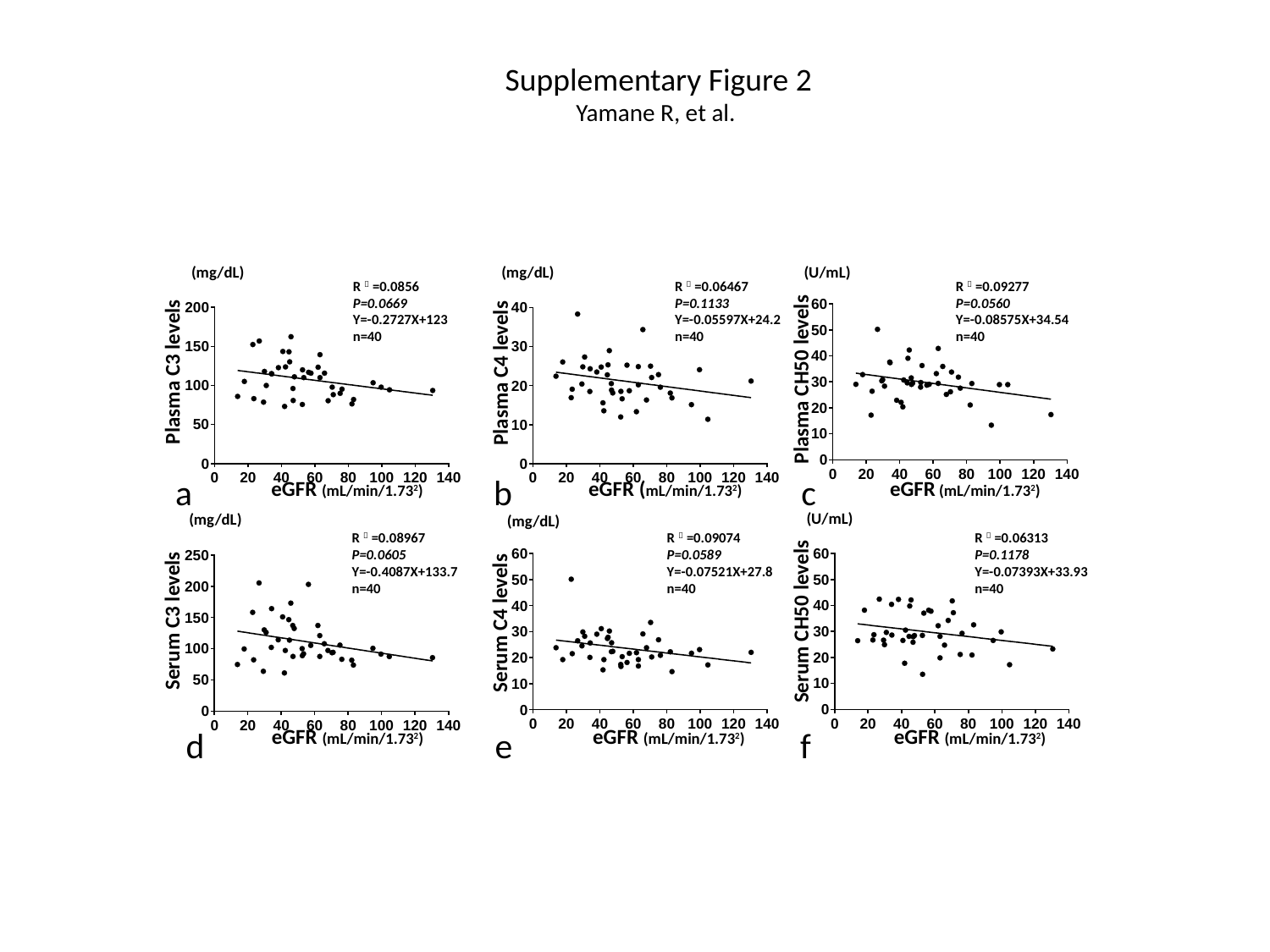

Supplementary Figure 2
Yamane R, et al.
(mg/dL)
(mg/dL)
(U/mL)
R２=0.0856
P=0.0669
Y=-0.2727X+123
n=40
R２=0.06467
P=0.1133
Y=-0.05597X+24.2
n=40
R２=0.09277
P=0.0560
Y=-0.08575X+34.54
n=40
Plasma C3 levels
Plasma C4 levels
Plasma CH50 levels
a
b
c
eGFR (mL/min/1.732)
eGFR (mL/min/1.732)
eGFR (mL/min/1.732)
(U/mL)
(mg/dL)
(mg/dL)
R２=0.08967
P=0.0605
Y=-0.4087X+133.7
n=40
R２=0.09074
P=0.0589
Y=-0.07521X+27.8
n=40
R２=0.06313
P=0.1178
Y=-0.07393X+33.93
n=40
Serum C3 levels
Serum CH50 levels
Serum C4 levels
d
e
f
eGFR (mL/min/1.732)
eGFR (mL/min/1.732)
eGFR (mL/min/1.732)

## Slide 3
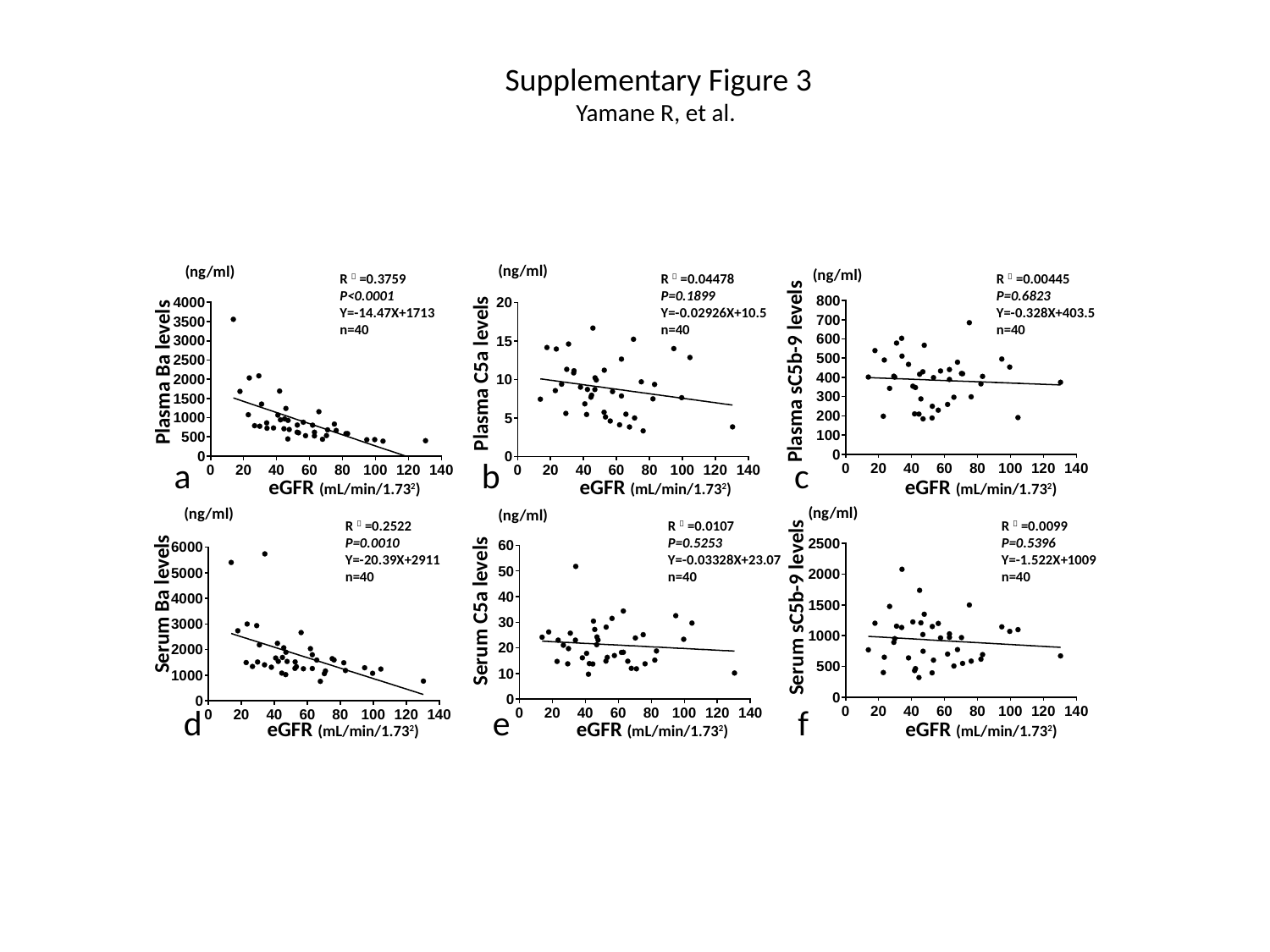

Supplementary Figure 3
Yamane R, et al.
(ng/ml)
(ng/ml)
(ng/ml)
R２=0.3759
P<0.0001
Y=-14.47X+1713
n=40
R２=0.04478
P=0.1899
Y=-0.02926X+10.5
n=40
R２=0.00445
P=0.6823
Y=-0.328X+403.5
n=40
Plasma sC5b-9 levels
Plasma Ba levels
Plasma C5a levels
a
b
c
eGFR (mL/min/1.732)
eGFR (mL/min/1.732)
eGFR (mL/min/1.732)
(ng/ml)
(ng/ml)
(ng/ml)
R２=0.2522
P=0.0010
Y=-20.39X+2911
n=40
R２=0.0107
P=0.5253
Y=-0.03328X+23.07
n=40
R２=0.0099
P=0.5396
Y=-1.522X+1009
n=40
Serum Ba levels
Serum sC5b-9 levels
Serum C5a levels
d
e
f
eGFR (mL/min/1.732)
eGFR (mL/min/1.732)
eGFR (mL/min/1.732)
