# Supplementary material for: Serum and plasma levels of Ba, but not those of soluble C5b-9, might be affected by renal function in chronic kidney disease patients
Source: BMC Nephrol. 2023 Feb 2;24:26. doi: 10.1186/s12882-022-03022-z (PMC9893599; doi:10.1186/s12882-022-03022-z)
Supplement: Supplementary file 2 — Additional file 2: Supplementary Table 1. Sample dilutions for ELISA assays in this study. [file 12882_2022_3022_MOESM2_ESM.pdf]

Supplementary Table 1 Sample dilutions for ELISA assays in this study

ELISA assay	Dilution of serum samples	Dilution of plasma samples
C3	×30~×60	×30~×60
C4	×30~×50	×30~×50
Ba	×2000*~×4000	×1000*~×2000
C5a	×50*	×20*
Soluble C5b-9	×50~×100	×20~×50

* Recommended dilution from information sheet of each ELISA kit
